# Stability of the *Plasmodium falciparum* AMA1-RON2 Complex Is Governed by the Domain II (DII) Loop

**DOI:** 10.1371/journal.pone.0144764

**Published:** 2016-01-05

**Authors:** Roberto F. Delgadillo, Michelle L. Parker, Maryse Lebrun, Martin J. Boulanger, Dominique Douguet

**Affiliations:** 1 Institut de Pharmacologie Moléculaire et Cellulaire, Université de Nice Sophia-Antipolis, CNRS, UMR 7275, 660, route des Lucioles, Sophia Antipolis, 06560, Valbonne, France; 2 Department of Biochemistry & Microbiology, University of Victoria, PO Box 3055 STN CSC, Victoria, BC, V8W 3P6, Canada; 3 UMR 5235 CNRS, Université de Montpellier, 34095, Montpellier, France; Monash University, AUSTRALIA

## Abstract

*Plasmodium falciparum* is an obligate intracellular protozoan parasite that employs a highly sophisticated mechanism to access the protective environment of the host cells. Key to this mechanism is the formation of an electron dense ring at the parasite-host cell interface called the Moving Junction (MJ) through which the parasite invades. The MJ incorporates two key parasite components: the surface protein Apical Membrane Antigen 1 (AMA1) and its receptor, the Rhoptry Neck Protein (RON) complex, the latter one being targeted to the host cell membrane during invasion. Crystal structures of AMA1 have shown that a partially mobile loop, termed the DII loop, forms part of a deep groove in domain I and overlaps with the RON2 binding site. To investigate the mechanism by which the DII loop influences RON2 binding, we measured the kinetics of association and dissociation and binding equilibria of a *Pf*RON2sp1 peptide with both *Pf*AMA1 and an engineered form of *Pf*AMA1 where the flexible region of the DII loop was replaced by a short Gly-Ser linker (ΔDII-*Pf*AMA1). The reactions were tracked by fluorescence anisotropy as a function of temperature and concentration and globally fitted to acquire the rate constants and corresponding thermodynamic profiles. Our results indicate that both *Pf*AMA1 constructs bound to the *Pf*RON2sp1 peptide with the formation of one intermediate in a sequential reversible reaction: A↔B↔C. Consistent with Isothermal Titration Calorimetry measurements, final complex formation was enthalpically driven and slightly entropically unfavorable. Importantly, our experimental data shows that the DII loop lengthened the complex half-life time by 18-fold (900 s and 48 s at 25°C for *Pf* and ΔDII-*Pf* complex, respectively). The longer half-life of the *Pf* complex appeared to be driven by a slower dissociation process. These data highlight a new influential role for the DII loop in kinetically locking the functional binary complex to enable host cell invasion.

## Introduction

Parasites in the phylum Apicomplexa include the etiological agents of malaria and toxoplasmosis. Malaria is a major health problem in much of the tropical and subtropical countries with an estimated 207 million cases in 2012 and 627,000 deaths, most of them children [1]. Amongst the 5 malaria species that affect humans, *Plasmodium falciparum* (*Pf*) is the most deadly form. Despite continuous efforts in vaccine development, prevention of malaria remains difficult and the spread of drug-resistant parasites highlights the critical need for new antimalarial strategies [2].

The invasion machinery is highly conserved in apicomplexan parasites and involves a structure called the moving junction (MJ) formed between the apex of the parasite and the host cell membrane [3]. The MJ anchors the parasite to the host cell membrane and allows its active internalization into a protective parasitophorous vacuole (PV). The MJ is initiated by injection of the Rhoptry Neck (RON) complex into the host cell, where RON2 spans the membrane and functions as a receptor for the Apical Membrane Antigen 1 (AMA1) protein located on the parasite surface [4–7]. It was recently reported that these proteins are essential for host cell invasion by *P*. *falciparum* and *T*. *gondii* [8, 9]. *Plasmodium spp*. encodes single copies of AMA (AMA1) and RON2 whose interaction is critical for invasion. Antibodies or peptides that prevent formation of the AMA1-RON2 complex also block invasion [10–17]. Therefore, disrupting the AMA1-RON2 complex offers new strategies for the development of anti-infectives.

Structural studies of ectodomains of AMA1 revealed a stacked three-domain architecture (DI, DII and DIII). Domains I and II are closely associated PAN domains that form a long and conserved hydrophobic trough partially occupied by a loop extending from domain II called DII loop [11, 12, 18, 19]. Analysis of AMA1 structures in apo and complex forms with antibodies or peptide inhibitors revealed that the DII loop is flexible and can adopt multiple conformations [11–13, 18–23]. In *Pf*AMA1, the DII loop encompasses residues 346 to 395 (50 amino acids) that undergoes a substantial conformational change to reveal the binding site for *Pf*RON2 [21]. Indeed, the crystal structure of *P*. *falciparum* AMA1 in complex with a 39-mer [2021–2059] *Pf*RON2 peptide (sp) showed that the DII loop was disordered [23]. Notably, this small *Pf*RON2sp synthetic peptide is able to compete in the nanomolar range with the native RON2 for AMA1 interaction *in vivo* [23].

The absence of polymorphisms in the DII loop suggests an important function for this substructure [24]. Recently, Parker *et al*. reported that the DII loop in *T*. *gondii* is able to regulate AMA1 selectivity for its cognate RON2 by competitive binding [25]. In this study, authors engineered a ΔDII-loop form of *Tg*AMA1 where the DII loop (residues 333–369) was replaced by a short Gly-Ser linker. This truncated form of *Tg*AMA1 preserved a similar affinity for *Tg*RON2 [25]. In the present study, we investigated the kinetics and the thermodynamics of the interaction between *Pf*RON2sp1 and both native and ΔDII-loop forms of *Pf*AMA1. In contrast to *T*. *gondii*, the ΔDII-loop form of *Pf*AMA1 exhibits a 2-fold weaker affinity for its *Pf*RON2sp synthetic peptide. Our results provide the kinetic and thermodynamic rationale for a stabilizing role of the DII loop in *P*. *falciparum* AMA1-RON2 complex.

## Materials and Methods

### Peptides Synthesis (Table 1)

Unlabeled and fluorescein-labeled (F*) peptides corresponding to residues 2021–2059 of *Pf*RON2sp1 were purchased from Kinexus (Vancouver, Canada) with a purity ≥ 95% and were disulfide cyclized. The labeled peptide is referred as F*PfRON2sp1.

**Table 1:** *Pf*RON2sp synthetic peptides. Peptides were cyclized at the cysteine residues (bold). Unlabeled peptides were used for the complex dissociation experiments. F*: 5-carboxyfluorescein (5-FAM).

Peptide name	Sequence
*Pf*RON2sp1 (39 aa)[2021–2059]	DITQQAKDIGAGPVAS**C**FTTRMSPPQQI**C**LNSVVNTALS
F*PfRON2sp1 (39 aa)[2021–2059]	5-FAM-DITQQAKDIGAGPVAS**C**FTTRMSPPQQI**C**LNSVVNTALS

### *Pf*AMA1 recombinant protein production

A codon-optimized gene encoding DI-DII of *Pf*AMA1 3D7 [26] (residues 104–438; numbering based on the initiation methionine in the signal sequence, PF11_0344) was synthesized by GenScript and subcloned into a modified pAcGP67B vector (Pharmingen) for expression in insect cells using established protocols [22]. For the ΔDII-*Pf*AMA1 construct, the segment KQYEQHLTDYEKIKEGFKNKNASMIKSAFLPTGAFKA (residues 351 to 387) within the *Pf*AMA1 DI-DII construct was replaced with 7 Gly/Ser residues, and Ser/Thr residues in predicted N-linked glycosylation sites (NxS/T) were mutated to Ala. Proteins were purified by Ni-affinity chromatography, cleaved with thrombin to remove the hexa-histidine tag, further purified by size exclusion chromatography, and concentrated to between 4.5 and 20 mg/mL. Final yield of recombinant protein was between 3 and 20 mg per L of culture.

### *Pf*RON2D3 production

A sequence encoding a portion of *Pf*RON2D3 (amino acids Asp2021 to Ser2059) was synthesized and subcloned into a modified pET32a vector (Novagen) incorporating N-terminal hexahistidine and thioredoxin (TRX) tags with a thrombin cleavage site. The fusion protein was produced in *E*. *coli* BL21 cells. For ITC experiments, thioredoxin fusion of *Pf*RON2D3 was produced in *E*. *coli* BL21 cells and purified by nickel-affinity and SEC.

### Isothermal Titration Calorimetry

Purified *Pf*AMA1, ΔDII-*Pf*AMA1, and *Pf*RON2D3-TRX were dialyzed against ITC buffer (20 mM Hepes pH 7.5, 150 mM NaCl) at 4°C. All ITC experiments were carried out at 25°C on a MicroCal iTC200 instrument. The sample cell contained *Pf*AMA1 or ΔDII-*Pf*AMA1 (10 μM), and *Pf*RON2D3-TRX (110 μM) was added in 17 injections of 2.2 μL each. For TRX-fused peptide, TRX was injected as a negative control and showed no detectable binding. Data were processed using Origin software (MicroCal) and the dissociation constants (K_d_) were determined using a one binding site model.

### Equilibrium Titrations and Kinetic Studies of *Pf*AMA1-*Pf*RON2sp1

All measurements were carried out in PBS buffer at pH of 7.4 with at least 0.1% BSA (Bovine Serum Albumin) on 96-well, black, flat microplates, made of a nonbinding surface (NBS) polystyrene nonionic hydrophilic surface (Cat No. 3993; Corning, Amsterdam, Netherlands).

Data acquisition was carried out on a Wallac 2103 HTS Microplate EnVision Reader, PerkinElmer (Wallac Oy) operated by Wallac EnVision Manager 1.12. The anisotropy (r) and total fluorescence (F) values were measured and stored in spreadsheets (S1 Text). The instrument was equipped with an: EnVision-2102 temperature control; Hamamatsu photomultiplier tube (R10130-10, serial No. AP7666); UV-Xenon fast tube lamp with spectral range 230–1100 nm; an optical module (481 FITC FP D505fp/D535, diameter 150 mm); 480 nm excitation filter (X480, 30 nm bandwidth, 70% T, 150 mm diameter); 535 nm S orientated emission filter (40 nm bandwidth, 80% T, 150 mm diameter); 535 nm P orientated emission filter (40 nm bandwidth, 80%T, 150 mm diameter). Titration scans were carried out by row-bi-directional, 10 flashes, 5–6 mm height, with a G factor of 1.04 and 750 PMT gain.

All experiments were carried out at 20°C, 25°C and 30°C with the temperature measured inside the wells with a thermocouple VWR traceable® (-200°C to 1370°C) with an error range of +/- 0.1°C. Long incubation times were allowed for uniformed temperature distribution of the plates for each of the 96 wells.

#### Binding titration experiments

Binding titration experiments were carried out with 10 nM of fluorescein labeled probe titrated with an increasing amount of protein (0 to 10 μM). Titration data was fitted by a nonlinear regression model embed in a simplex minimization routine solving the quadratic version of Eq 1 (S2 Text) where *K*_*d*_ is the dissociation constant, Y is the fraction saturation, P is the free protein, X_T_ is the total labeled peptide (F*PfRON2sp1) and P_T_ is the total protein [27]:

$$Y=\frac{\left(\frac{1}{K_{d}}\right)\left(P_{T}-YX_{T}\right)}{1+\left(\frac{1}{K_{d}}\right)\left(P_{T}-YX_{T}\right)}$$ (1)

#### Association and dissociation kinetics

The association reactions were acquired with a final concentration of 10 nM of the labeled probe and 50, 100 and 200 nM of *Pf*AMA1 and ΔDII-*Pf*AMA1 proteins for concentration dependence experiments. The dissociation reactions were carried out with 10 nM of the preformed complex challenged with the unlabeled ligand (*Pf*RON2sp1) at concentrations of 2500 nM and 5000 nM, for a 250× and 500× excess, respectively. A total of 48 association reactions and 12 dissociation reactions were collected at 20, 25 and 30°C, with data points collected at interval of 6 seconds for at least 10–15 minutes and stored in spreadsheets as a function of time. The kinetic traces were well described by double-exponential functions (S1 Table). Data sets were used in the global fitting step described below in order to propose the most probable reaction mechanism.

#### Global fitting procedure to calculate rate constants and reaction models

The association and dissociation kinetic data sets were fitted globally to extract rate constants (k) as a function of temperature according to the Arrhenius relationship (Eq 2) where E_a_ is the activation energy, A is the pre-exponential factor, T is the temperature in Kelvin degrees and R is the gas constant (1.987 cal K^-1^ mol^-1^).

k=Ae−Ea(RT) (2)

We used the global fitting algorithm [27] to evaluate rate constants according to three reaction mechanisms: 1) A simple reversible reaction model (Eq 3) with two rate constants (k_1_, k_2_), a transition state (‡) and two activation energies (Ea_1_ and Ea_2_) for forward and reverse reactions.

$$AMA1+F\;*\;PfRON2sp1\overset{k1}{\underset{k2}{↔}}\text{Complex}\;(A+B\overset{k1}{\underset{k2}{↔}}\text{C})$$ (3)

2) A one-intermediate reversible reaction model (Eq 4) with two transition states (‡^1^ and ‡^2^), four rate constants (k_1_ to k_4_) and four activation energies (E_a1_ to E_a4_).

$$AMA1+F\;*\;PfRON2sp1\overset{k1}{\underset{k2}{↔}}I\overset{k3}{\underset{k4}{↔}}Complex\;(A+B\overset{k1}{\underset{k2}{↔}}C\overset{k3}{\underset{k4}{↔}}D)$$ (4)

3) A unimolecular conversion between an inactive (A) and an active (A*) state of the protein (Eq 5).

$$AMA1_{Inactive}\overset{k1}{\underset{k2}{↔}}AMA1_{Active}+F\;*\;PfRON2sp1\overset{k3}{\underset{k4}{↔}}\text{Complex}\;(A_{Inactive}\overset{k1}{\underset{k2}{↔}}A_{Active}^{*}+B\overset{k3}{\underset{k4}{↔}}\text{C})$$ (5)

The differential equations for the simplest model (Eq 3) is presented in the following matrix form (Eq 6) [28] which contains all species involved in the reaction such as unlabeled proteins (A), labeled peptide (B, F*PfRON2sp1) and complex (C, e.g. *Pf*AMA1-F*PfRON2sp1). The kinetic response function (Eq 7) includes the specie concentrations calculated with the matrix, the quantum yield (QY) and anisotropy values (r) of the fluorescent species [29]. The resulted rate constants let us calculate the K_d_ (Eq 8).

$$\left(\begin{array}{c} -dA/dt\\ -dB/dt\\ -dC/dt \end{array}\right)=\begin{array}{c} k_{1}AB\\ k_{1}AB\\ -k_{1}AB \end{array}\begin{array}{c} \;-k_{2}C\\ \;-k_{2}C\\ \;+k_{2}C \end{array}\;=\;\left(\begin{array}{c} k_{1}B\\ \;0\\ -k_{1}B \end{array}\begin{array}{c} \;0\\ \;k_{1}A\\ \;0 \end{array}\begin{array}{c} \;-k_{2}\\ \;-k_{2}\\ \;k_{2} \end{array}\right)\left(\begin{array}{c} A\\ B\\ C \end{array}\right)$$ (6)

$$r\left(t\right)=\sum \;x_{i}\left(t\right)QY_{i}r_{i}={\left(\frac{d\left(B\right)}{dt}QY_{B}r_{B}\right)}_{RON2}+{\left(\frac{d\left(C\right)}{dt}QY_{C}r_{C}\right)}_{Complex}$$ (7)

$$K_{d}=\frac{k_{2}}{k_{1}}$$ (8)

The differential equations for the second model with one intermediate (Eq 4) is shown in Eq 9 in which C is the fluorescent intermediate and D the final complex. The kinetic response function and the K_d_ calculation are shown in Eq 10 and Eq 11, respectively.

$$\left(\begin{array}{c} -dA/dt\\ -dB/dt\\ \begin{array}{c} -dC/dt\\ -dD/dt \end{array} \end{array}\right)=\;\begin{array}{c} \;k_{1}AB\\ -k_{1}AB\\ \begin{array}{c} -k_{1}AB\\ -k_{3}C \end{array} \end{array}\begin{array}{c} -k_{2}C\\ -k_{2}C\\ \begin{array}{c} \;(k_{2}+k_{3})\;C-k_{4}D\\ -k_{4}D \end{array} \end{array}=\left(\begin{array}{c} k_{1}B\\ 0\\ \begin{array}{c} -k_{1}B\\ 0 \end{array} \end{array}\;\begin{array}{c} 0\\ k_{1}A\\ \begin{array}{c} 0\\ 0 \end{array} \end{array}\begin{array}{c} -k_{2}\\ -k_{2}\\ \begin{array}{c} \;\left(\;k_{2}+k_{3}\right)\;\\ -k_{3} \end{array} \end{array}\begin{array}{c} 0\\ 0\\ \begin{array}{c} -k_{4}\\ k_{4} \end{array} \end{array}\right)\left(\begin{array}{c} A\\ B\\ \begin{array}{c} C\\ D \end{array} \end{array}\right)$$ (9)

$$r\left(t\right)=\sum \;x_{i}\left(t\right)QY_{i}r_{i}={\left(\frac{d\left(B\right)}{dt}QY_{B}*r_{B}\right)}_{RON2}+{\left(\frac{d\left(C\right)}{dt}QY_{C}*r_{C}\right)}_{I}+{\left(\frac{d\left(D\right)}{dt}QY_{D}*r_{D}\right)}_{Complex}$$ (10)

$$\frac{1}{K_{d}}=K_{a}=\frac{k_{1}}{k_{2}}+\left(\frac{k_{1}k_{3}}{k_{2}k_{4}}\right)$$ (11)

The differential equations for the third model representing a conversion between an inactive (A) to an active state (A*) of the protein (Eq 5) is shown in Eq 12 in which B is the labeled peptide and C is the final complex. The kinetic response function and the K_d_ calculation are shown in Eq 13 and Eq 14, respectively.

$$\left(\begin{array}{c} -dA/dt\\ -dA^{*}/dt\\ \begin{array}{c} -dB/dt\\ -dC/dt \end{array} \end{array}\right)=\;\begin{array}{c} \;k_{1}A\\ -k_{1}A\\ \begin{array}{c} k_{3}BA^{*}\\ -k_{3}BA^{*} \end{array} \end{array}\begin{array}{c} -k_{2}A^{*}\\ +k_{2}A^{*}+k_{3}BA^{*}-k_{4}\;C\\ \begin{array}{c} -k_{4}C\\ k_{4}C \end{array} \end{array}\;=\left(\begin{array}{c} k_{1}\\ -k_{1}\\ \begin{array}{c} 0\\ 0 \end{array} \end{array}\;\begin{array}{c} -k_{2}\\ k_{2}\\ \begin{array}{c} 0\\ 0 \end{array} \end{array}\begin{array}{c} 0\\ -k_{3}A^{*}\\ \begin{array}{c} \;k_{3}A^{*}\;\\ -k_{3}A^{*} \end{array} \end{array}\begin{array}{c} 0\\ -k_{4}\\ \begin{array}{c} -k_{4}\\ k_{4} \end{array} \end{array}\right)\left(\begin{array}{c} A\\ A^{*}\\ \begin{array}{c} B\\ C \end{array} \end{array}\right)$$ (12)

$$r\left(t\right)=\sum \;x_{i}\left(t\right)QY_{i}r_{i}={\left(\frac{d\left(B\right)}{dt}QY_{B}*r_{B}\right)}_{RON2}+{\left(\frac{d\left(C\right)}{dt}QY_{C}*r_{C}\right)}_{complex}$$ (13)

$$\frac{1}{K_{d}}=K_{a}=\frac{k_{3}}{k_{4}}$$ (14)

#### Model evaluation

The global fitting procedure optimizes rate constants and activation energies by reducing the Residual Sum of Squared (RSS) between observed and calculated data points from association and dissociation experiments as well as K_d_ values at each temperature and concentration. In order to evaluate each model, we also calculated the Akaike selection criterion value (AIC) (Eq 15) [30, 31]. The AIC formula considers the number of estimated parameters *k’* (e.g. rate constants and activation energies), the global Residual Sum of Squared (RSS) and the number (N) of observations (S3 Text). The term *k’* adds a penalty to avoid an overfitting when the number of parameters increases. The lowest AIC value indicates the most probable model to describe the observed data.

$$AIC=NLog\left(\frac{RSS_{Global}}{N}\right)+\;2\;k\prime$$ (15)

#### Thermodynamics of the transition states (‡)

ΔH^‡^ ΔS^‡^ and ΔG^‡^ can be acquired by applying Eq 16 and the Eyring equation (Eq 17) [32] where *k* is the rate constant, k_B_ is the Boltzmann’s constant (3.3 x 10^−24^ cal K^-1^) and *h* is the Planck’s constant (1.58 x 10^−34^ cal s).

$$\Delta H^{‡}=E_{a}-RT$$ (16)

$$k=\frac{k_{B}T}{h}exp\;\left(-\frac{\Delta G^{‡}}{RT}\right)=\frac{k_{B}T}{h}exp\;\left(\frac{\Delta S^{‡}}{R}\right)\;exp\;\left(-\frac{\Delta H^{‡}}{RT}\right)$$ (17)

#### Overall thermodynamic parameters

Overall thermodynamic parameters were calculated by two approaches: 1) by the summation of the transition-state parameters which were obtained by the global fitting of the kinetic data (Eq 16 and Eq 17). For example, the enthalpy cycle ΔΔH° was calculated by using enthalpies of activation (Eq 18). A similar summation can be carried out for the entropy ΔΔS° and for the free energy ΔΔG°.

$$\Delta \Delta {\text{H}}^{{}^{\circ}}=\sum \Delta {\text{H}}_{\text{forward}}^{⧧}-\sum \Delta {\text{H}}_{\text{reverse}}^{⧧}$$ (18)

2) by Isothermal Titration Calorimetry (ITC) experiments.

## Results

### The DII loop promotes a higher affinity interaction between *Pf*AMA1 and *Pf*RON2sp1 peptide

**Fig 1:**
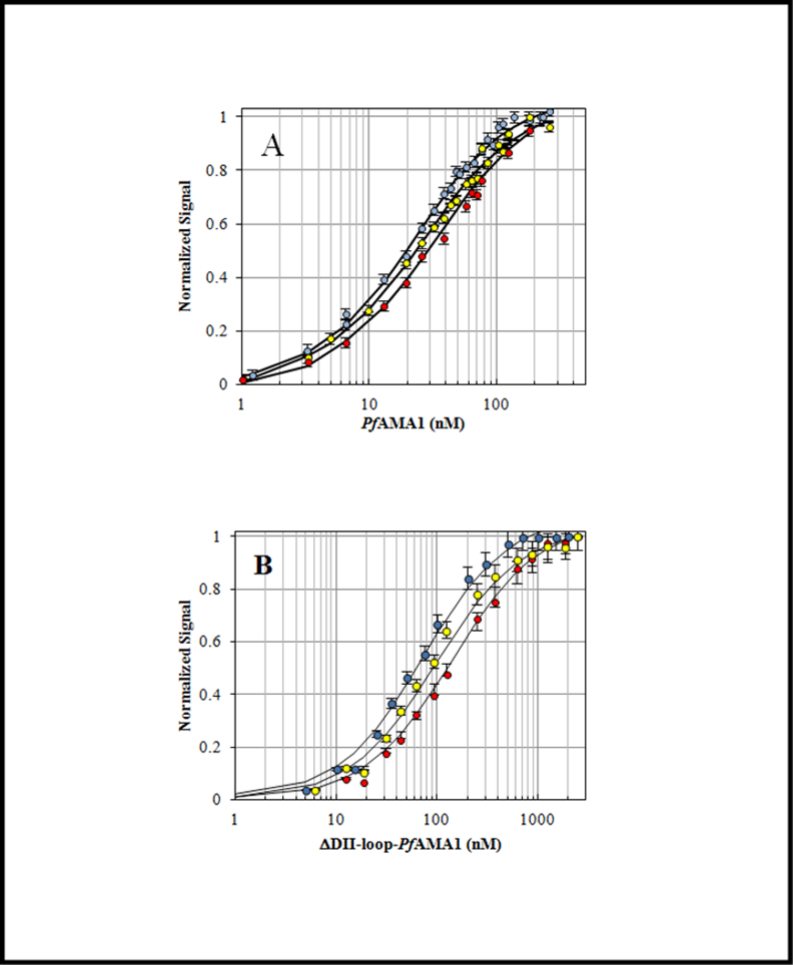
Titration experiments: A) *Pf*AMA1-F*PfRON2sp1, B) ΔDII-*Pf*AMA1-F*PfRON2sp1 at 20°C (blue), 25°C (yellow) and 30°C (red) titrated with an increasing amount of protein and 10 nM of labeled peptide. Solid lines represent the best fit (method in S2 Text). Error bars represent one standard deviation in the fluorescence-based experiments.

**Fig 2:**
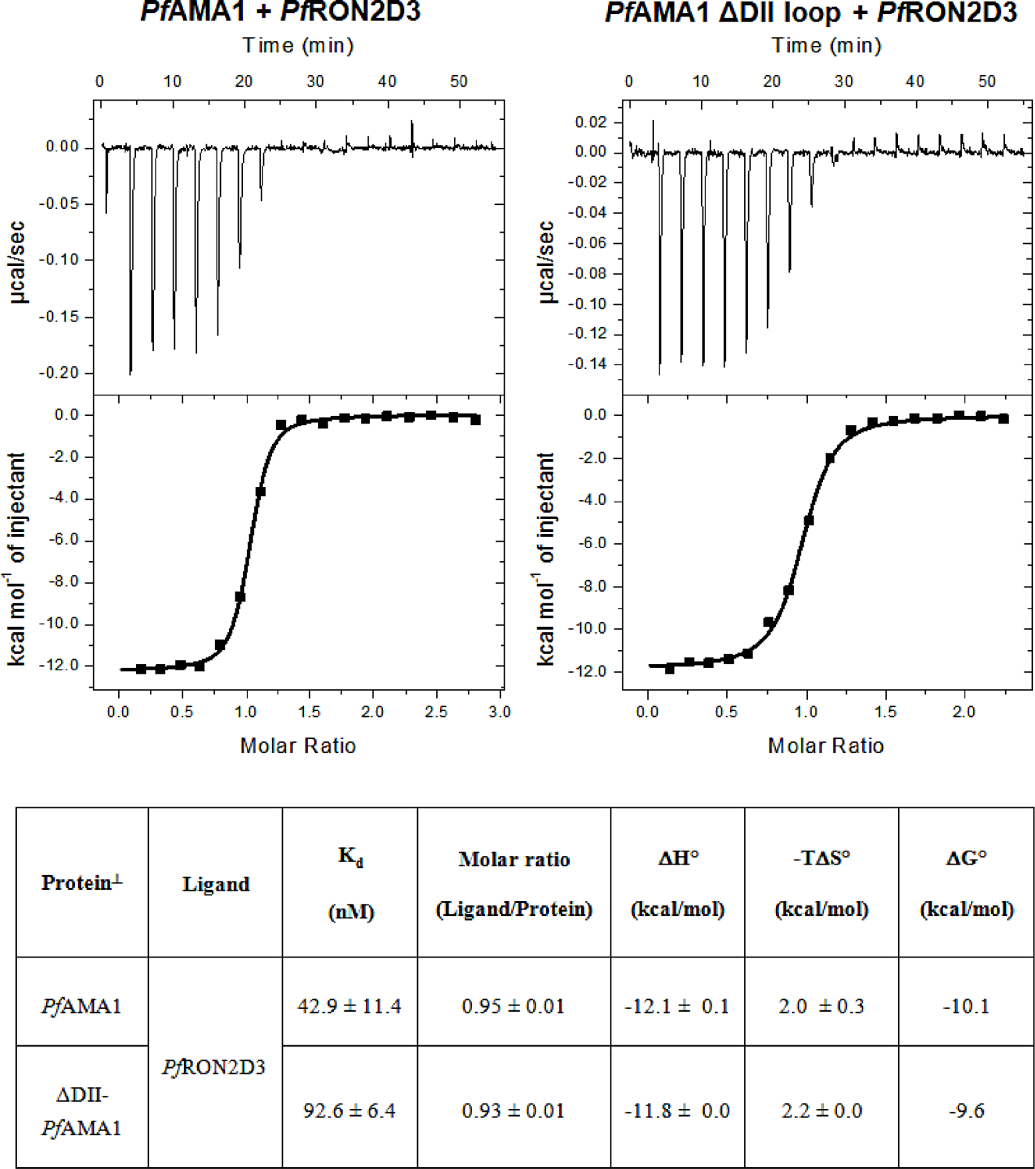
Isothermal Titration Calorimetry at 25°C. A constant amount of protein (10 μM) was titrated with an increasing amount of *Pf*RON2D3-TRX. Tabulated results represent the average of two experiments with standard deviation.

A ΔDII-*Pf*AMA1 recombinant protein was constructed by replacing the segment KQYEQHLTDYEKIKEGFKNKNASMIKSAFLPTGAFKA (residues 351 to 387) within the *Pf*AMA1 DI-DII construct with 7 Gly/Ser residues. Then, the equilibrium binding studies of *Pf*AMA1 and ΔDII-*Pf*AMA1 complexes were investigated by the change of fluorescence anisotropy upon binding (Fig 1) and by Isothermal Titration Calorimetry (ITC) (Fig 2). The anisotropy binding titrations were acquired with an increasing amount of unlabeled protein added to a constant amount of labeled peptide at 20°C, 25°C and 30°C. *Pf*AMA1 bound to *Pf*RON2sp1 peptide with an affinity of 42 nM, consistent with previously published SPR measurement (Table 2) [23]. These results correlated well with the data obtained from ITC experiments for *Pf*RON2sp1 binding to *Pf*AMA1 and ΔDII-*Pf*AMA1 at 25°C (Fig 2 and Table 2). The truncated form ΔDII-*Pf*AMA1 showed a 2-fold weaker affinity to the same *Pf*RON2sp1 (92 nM). These results suggested an intriguing mechanism whereby the *Pf*AMA1 DII loop enables a tighter interaction with *Pf*RON2sp1.

**Table 2:** Comparison of equilibrium dissociation constants (K_d_) of complexes.

Complex	Anisotropy Titration^¶^ K_d_ (nM)			Complex	ITC^¶^K_d_ (nM)	SPRK_d_ (nM)
	20°C	25°C	30°C		25°C	Room temp
*Pf*AMA1-F*PfRON2sp1	31.0 ± 2	43 ± 3	61.0 ± 5	*Pf*AMA1-*Pf*RON2D3	42.9 ± 11.4	20.3 ±6.3[23]
ΔDII-*Pf*AMA1-F*PfRON2sp1	60 ± 5	94.0 ± 5	125 ± 10	ΔDII-*Pf*AMA1-*Pf*RON2D3	92.6 ± 6.4	ND

^**¶**^Anisotropy titrations were carried out with an increasing amount of protein added to a constant amount of labeled probe (10 nM). In contrast, Isothermal Titration Calorimetry (ITC) experiments were carried out with a constant amount of protein and an increasing amount of peptide. ND: not determined.

### The DII loop affects the binding kinetics

**Fig 3:**
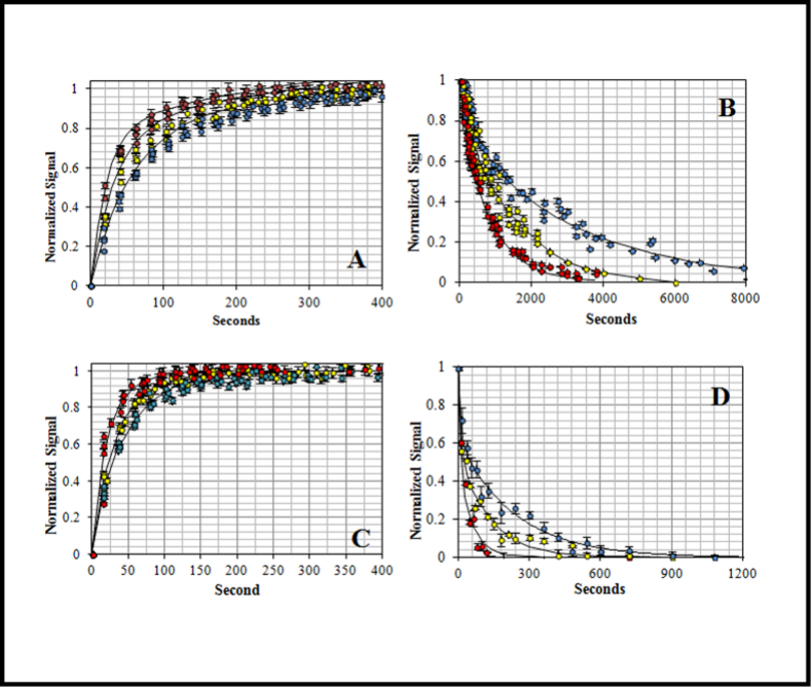
Temperature dependence of association and dissociation kinetics: *Pf*AMA1-F*PfRON2sp1 (A, B) and ΔDII-*Pf*AMA1-F*PfRON2sp1 (C, D) at 20°C (blue), 25°C (yellow) and 30°C (red). The association (A and C) and dissociation (B and D) reaction rates increased as temperature increases accordingly to positive activation energies. Complex dissociation curves were acquired by challenging a preformed complex (10 nM) with unlabeled *Pf*RON2sp1 (500x excess). The solid black lines are the globally fitted data according to the one-intermediate model (Eq 4). Error bars represent one standard deviation in the fluorescence-based experiments.

To probe the underlying mechanism by which the DII loop contributes to RON2 binding, we measured the association kinetics of both *Pf*AMA1 and ΔDII-*Pf*AMA1 with the peptide and dissociation for both *Pf* and ΔDII-*Pf* complex when challenged with unlabeled *Pf*RON2sp1 peptide. In all cases, these association and dissociation experiments showed a biphasic behavior with a fast and a slow phase (Fig 3) suggesting an elaborate reaction mechanism (Eq 4 or Eq 5) instead of a simple and reversible mechanism (Eq 3).

**Fig 4:**
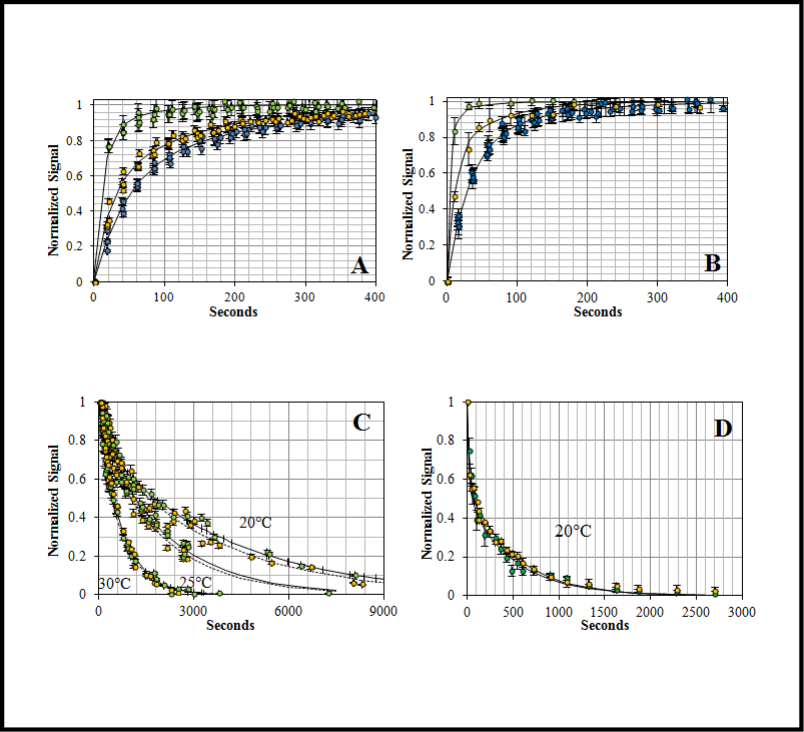
Concentration dependence of the association and dissociation kinetics: Association: A) *Pf*AMA1 at 50 nM (blue), 100 nM (gold) and 200 nM (green) reacting with F*PfRON2sp1 (10 nM). B) ΔDII-*Pf*AMA1 at 50 nM (blue), 100 nM (gold) and 200 nM (green) reacting with F*PfRON2sp1 (10 nM). The reaction speed increased proportionally to protein concentration. The curves showed a biphasic behavior suggesting the presence of a two-step reaction mechanism. Dissociation: C) *Pf*AMA1-F*PfRON2sp1 at three temperatures (20°C, 25°C and 30°C) and D) ΔDII-*Pf*AMA1-F*PfRON2sp1 at 20°C challenged with unlabeled *Pf*RON2sp1 at 2500 nM (250x excess, gold, solid line) and 5000 nM (500x excess, green, dashed line). Both complex dissociations showed no concentration dependence. The concentration independence indicated the existence of a unimolecular process where the unlabeled peptide waits for the spontaneous leaving of the labeled peptide (F*PfRON2sp1) from the binding site. Error bars represent one standard deviation in the fluorescence-based experiments.

**The association reactions** were collected at 20°C, 25°C and 30°C (Fig 3A and 3C) to acquire the forward association rate constants (k_on_) as a function of temperature. In all cases, the reaction signal increased as the reaction progressed and the complex was formed. The kinetic traces were initially analyzed using a double-exponential function (S1 Table). The association reactions showed a proportional increase in the rate when *Pf*AMA1 and ΔDII-*Pf*AMA1 concentrations increased from 50 nM to 200 nM for a ratio of 5×, 10× and 20× with respect to the F*PfRON2sp1 concentration (Fig 4A and 4B). These data are consistent with a bimolecular reaction where the rate constants depend on the concentration of both protein and peptide reactants. Importantly, the observed homogeneity and linearity of the plots indicated that aggregation effects can be excluded under these reaction conditions.

**The dissociation experiments** of the preformed complexes were carried out at the same temperatures (20, 25 and 30°C) using the unlabeled *Pf*RON2sp1 peptide as the competing ligand in order to track the dissociation rate constants (k_off_) (Fig 3B and 3D). As the labeled peptide was replaced in the complex, the signal decreased as a function of time. For both complexes, the k_off_ increased with increasing temperature. Interestingly, despite increasing the amount of unlabeled peptide (250× and 500×), the speed of the reaction remained unchanged for *Pf* and ΔDII-*Pf* complexes (Fig 4C and 4D). The concentration-independence of the dissociation suggested a unimolecular mechanism of dissociation, independent of colliding, in which the labeled-bound peptide must first leave the binding site before accommodating the unlabeled peptide.

### Determination of the most probable reaction mechanism that describes the kinetics

**Fig 5:**
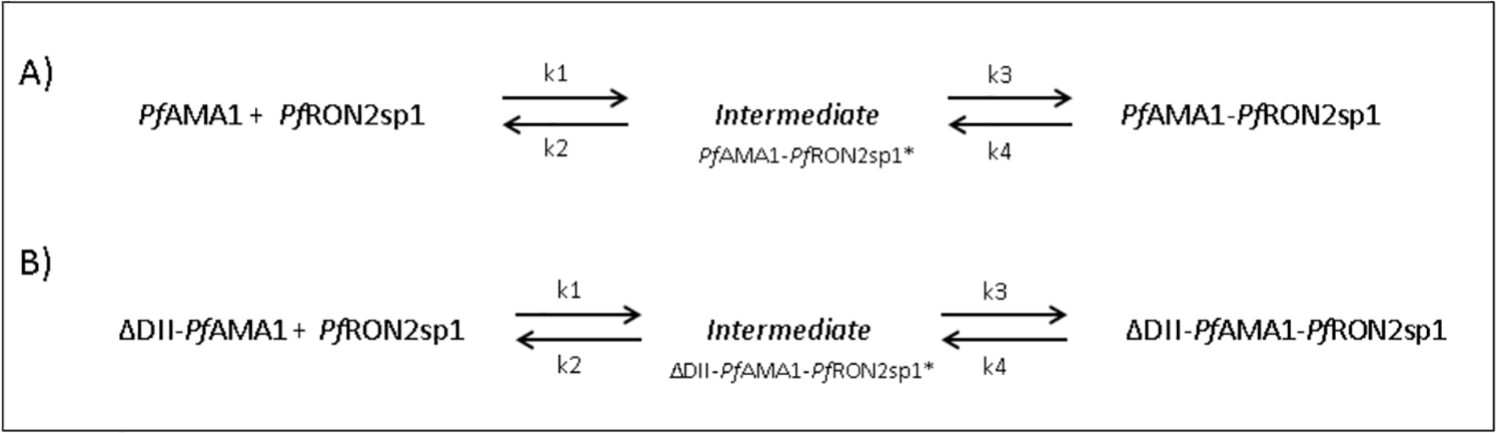
Two-step reaction mechanism with one-intermediate state (I) of *Pf*RON2sp1 peptide binding to A) *Pf*AMA1 and B) ΔDII-*Pf*AMA1.

The strong biphasic association and dissociation curves of the complexes are indicative of an elaborate reaction mechanism (Fig 3 and S1 Table). Nevertheless, we evaluated three different models: 1) a simple reversible model (Eq 3), 2) a one-intermediate model (Eq 4) and 3) a model that involves activation (conformational conversion) of the protein (Eq 5). The differential equations of these models were embedded in a simple minimization algorithm previously described [27] to obtain the rate constants simultaneously as a function of temperature and concentration. When comparing models, the Akaike selection criterion (AIC) value was in favor of the one-intermediate model (Table 3, S3 Text, S2 Fig and S3 Fig and Fig 5) for both *Pf*AMA1 and ΔDII-*Pf*AMA1 reactions (Table 4, Figs 3 and 4 (solid lines)). The selection of the one-intermediate model was also supported by the excellent agreement with experimental complex half-life, which is defined as the time required for 50% of the complex decay (Table 5).

**Table 3:** Model Evaluation. The most probable model has the lowest AIC value. The AIC criteria considers the goodness of the fit and places a penalty when the number of fitting parameters (k’) is increased (Eq 15). For F*PfRON2sp1 reacting with *Pf*AMA1 and ΔDII-*Pf*AMA1 proteins, the lowest value in both cases was achieved with the one-intermediate model.

Reaction	Model	k’	N*ln(RSS/N)	AIC
*Pf*AMA1 + F*PfRON2sp1	one-intermediate	8	-7.9	8.1
*Pf*AMA1 + F*PfRON2sp1	simple	4	14.1	22.1
*Pf*AMA1 + F*PfRON2sp1	interconversion	8	18.3	34.3
ΔDII-*Pf*AMA1 + F*PfRON2sp1	one-intermediate	8	-14.6	1.4
ΔDII-*Pf*AMA1 + F*PfRON2sp1	simple	4	7.5	15.5
ΔDII-*Pf*AMA1 + F*PfRON2sp1	interconversion	8	-5.4	10.6

**Table 4:** Calculated rate constants for *Pf* complexes for a one-intermediate model.

***Pf*AMA1-*Pf*RON2sp1**	**20°C**	**25°C**	**30°C**
**k**_**1**_ **(M**^**-1**^**s**^**-1**^**)**	73500 (± 9460)	114600 (± 12600)	176000 (± 21000)
**k**_**2**_ **(s**^**-1**^**)**	0.00478 (± 0.001)	0.00759 (± 0.001)	0.0119 (± 0.002)
**k**_**3**_ **(s**^**-1**^**)**	0.00041 (± 0.0001)	0.00053 (± 0.0002)	0.00069 (± 0.0002)
**k**_**4**_ **(s**^**-1**^**)**	0.0004 (± 0.0001)	0.00072 (± 0.0002)	0.00127(± 0.0004)
**k**_**1**_**/k**_**2**_ **(× 10**^**6**^ **M**^**-1**^**)**	15.4 (± 2.9)	15.1 (± 2.8)	14.8 (± 2.8)
**k**_**3**_**/k**_**4**_	1.0 (± 0.3)	0.7 (± 0.2)	0.5 (± 0.15)
**K**_**a**_ **(μM)**	30.9 (± 8)	26.2 (± 7)	22.9 (± 6)
**K**_**d**_ **(nM)**	32.3 (± 8)	38.2 (± 10)	43.8 (± 11)
**ΔDII-*Pf*AMA1-*Pf*RON2sp1**	**20°C**	**25°C**	**30°C**
**k**_**1**_ **(M**^**-1**^**s**^**-1**^**)**	78900 (± 6300)	116000 (± 9700)	168400 (± 15000)
**k**_**2**_ **(s**^**-1**^**)**	0.00417 (± 0.001)	0.0099 (± 0.001)	0.0229 (± 0.003)
**k**_**3**_ **(s**^**-1**^**)**	0.00459 (± 0.002)	0.0074 (± 0.003)	0.0117 (± 0.004)
**k**_**4**_ **(s**^**-1**^**)**	0.0812 (± 0.03)	0.1099 (± 0.04)	0.1473 (± 0.06)
**k**_**1**_**/k**_**2**_ **(× 10**^**6**^ **M**^**-1**^**)**	18.93 (± 2.5)	11.70 (± 1.6)	7.35 (± 1.0)
**k**_**3**_**/k**_**4**_	0.056 (± 0.01)	0.067 (± 0.02)	0.080 (± 0.02)
**K**_**a**_ **(μM)**	20.0 (± 6.0)	12.5 (± 3.8)	7.9 (± 2.4)
**K**_**d**_ **(nM)**	50.0 (± 15)	80.1 (± 24)	126.0 (± 38)

**Table 5:** Observed and calculated complex dissociation half-life.

Complex half-life^⊥^		20°C	25°C	30°C
*Pf*AMA1-F*PfRON2sp1	K_d_ (nM)	31.0 ± 2	43 ± 3	61.0 ± 5
*Pf*AMA1-F*PfRON2sp1	Experimental	1641 s (27.4 min)	900 s (15.0 min)	554 s (9.2 min)
*Pf*AMA1-F*PfRON2sp1	*Calculated*	*1730 s (28*.*8 min)*	*960 s (16*.*0 min)*	*544 s (9*.*1 min)*
**ΔDII-*Pf*AMA1-*Pf*RON2sp1**	K_d_ (nM)	60.0 ± 5	94.0 ± 5	125 ± 10
**ΔDII-*Pf*AMA1-*Pf*RON2sp1**	Experimental	100 s (1.6 min)	48 s (0.8 min)	30 s (0.5 min)
**ΔDII-*Pf*AMA1-*Pf*RON2sp1**	*Calculated*	*166 s (2*.*8 min)*	*69*.*9 s (1*.*2 min)*	*30 s (0*.*5 min)*

^**⊥**^ Observed complex half-life, t_1/2_ = ln2 /k_off slower_ [39]

### Calculation of the thermodynamic parameters

**Fig 6:**
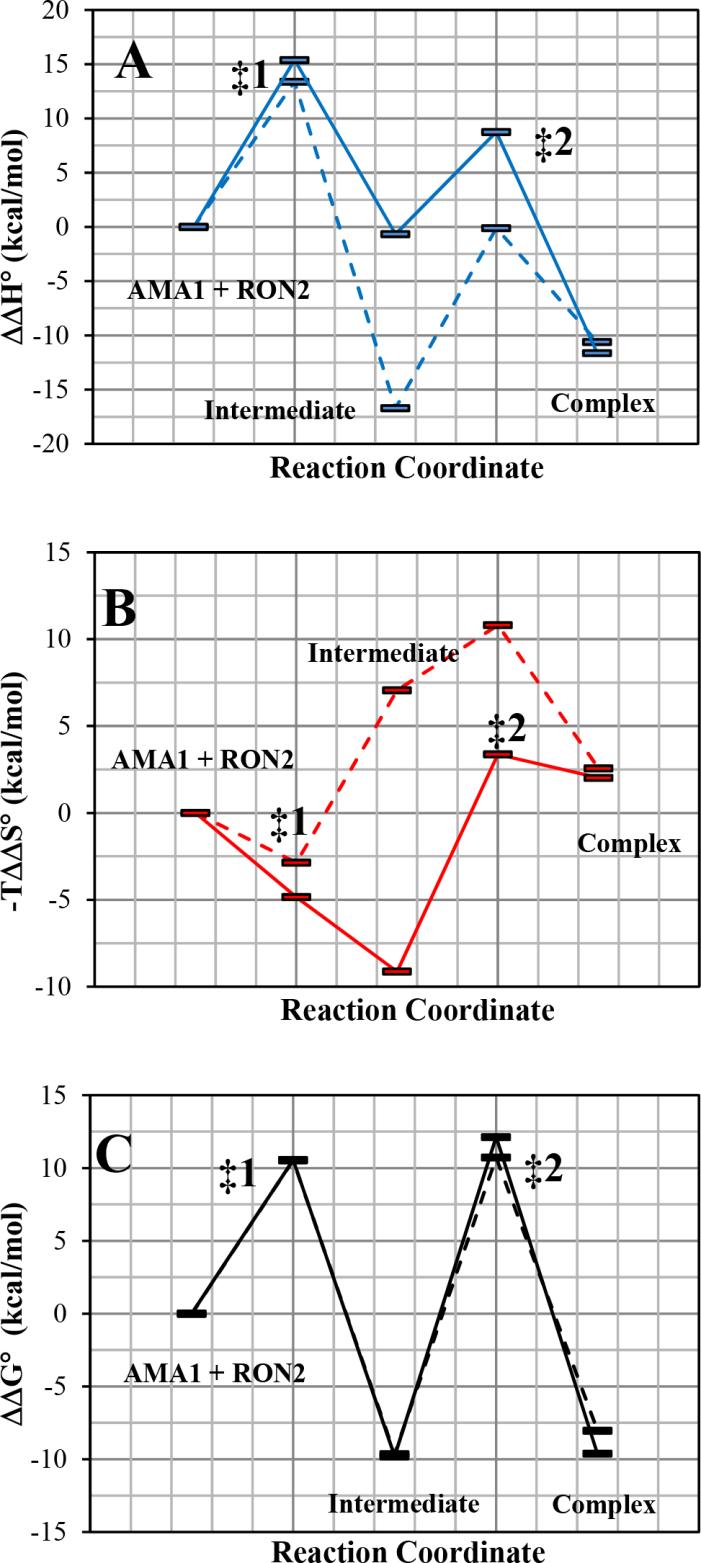
Thermodynamics profiles at 25°C, 1 M standard rate and 1 atm, A) ΔΔH°, B) -TΔΔS° and C) ΔΔG°: *Pf*AMA1-F*PfRON2sp1 (solid line) and ΔDII-*Pf*AMA1-F*PfRON2sp1 (slashed line). The free energies, enthalpies and entropies of the protein and peptide were set to zero.

The calculated thermodynamic parameters (Eq 16 and Eq 17) were in very good agreement with those obtained by ITC (Fig 2 and Table 6). Here, the enthalpy of activation (ΔH°^‡^) was calculated using the globally fitted Arrhenius temperature-dependent rate constants (Eq 2 and Eq 16) and the Gibbs free energy of activation (ΔG°^‡^) was calculated using the Eyring’s equation (Eq 17) to further deduce the entropy of activation (ΔS°^‡^). Finally, the global thermodynamic parameters ΔΔG°, ΔΔH° and -TΔΔS° of the reactions were calculated by the summation of the transition state energy values (Fig 6 and Table 6) [32]. Formation of both the *Pf*AMA1-*Pf*RON2sp1 and ΔDII-*Pf*AMA1-*Pf*RON2sp1 complexes were enthalpically driven but slightly entropically unfavorable (Fig 6 and Table 6) consistent with ITC results (Fig 2). Nevertheless, ΔDII-*Pf*AMA1 reaction with F*PfRON2sp1 differed both in enthalpy and entropy profiles as indicated their calculated values at transition and intermediate states in Fig 6 (slashed line) to those of *Pf*AMA1 reacting with the same peptide (solid line). Indeed, the intermediate (I) for *Pf* complex was entropically driven with a value of -TΔΔS°_(I)_ = -9.13 Kcal/mol with a small change in ΔΔH°_(I)_ = -0.67 Kcal/mol, whereas the intermediate (I) for ΔDII-*Pf* complex was enthalpically driven with a value of ΔΔH°_(I)_ = -16.7 Kcal/mol but with an unfavorable entropy value of -TΔΔS°_(I)_ = +7.06 Kcal/mol (Fig 6). These calculations indicate that despite the absence of the DII loop, which resulted in differences in entropy and enthalpy energies of the intermediate state, the same final complex is obtained where *Pf*RON2sp1 is anchored at both ends of the *Pf*AMA1 binding groove [25].

**Table 6:** Summary of the thermodynamic parameters for the binding of *Pf*RON2sp1 to *Pf*AMA1 and ΔDII-*Pf*AMA1. Calculations were carried out at 25°C, 1 M standard state and 1 atm according to the Eyring equation that relates rate constants with transition state thermodynamics [32].

Reaction	Free energy(Kcal/mol)	Enthalpy(Kcal/mol)	Entropy(cal/°Kmol)	-TΔS_i_°(Kcal/mol)
***Pf*AMA1-*Pf*RON2sp1**				
Reactants$\overset{{\text{k}}_{1}}{\to }$‡1	10.55	15.39	16.24	-4.84
Intermediate$\overset{{\text{k}}_{2}}{\to }$ ‡1	20.34	16.06	-14.37	4.29
Intermediate$\overset{{\text{k}}_{3}}{\to }$ ‡2	21.9	9.42	-41.91	12.5
Complex$\overset{{\text{k}}_{4}}{\to }$ ‡2	21.74	20.39	-4.53	1.35
**Overall** ^⊥^	**ΔΔG° = -9.63**	**ΔΔH° = -11.64**	**ΔΔS° = -6.77**	**-TΔΔS° = 2.02**
**ITC**	**ΔG° = -10.1**	**ΔH° = -12.1 ± 0.1**	**ΔS° = -6.7**	**-TΔS° = 2.0 ± 0.3**
**ΔDII-*Pf*AMA1-*Pf*RON2sp1**				
Reactants$\overset{{\text{k}}_{1}}{\to }$‡1	10.54	13.40	9.57	-2.85
Intermediate$\overset{{\text{k}}_{2}}{\to }$ ‡1	20.18	30.10	33.25	-9.91
Intermediate$\overset{{\text{k}}_{3}}{\to }$ ‡2	20.36	16.61	-12.59	3.75
Complex$\overset{{\text{k}}_{4}}{\to }$ ‡2	18.76	10.51	-27.68	8.25
**Overall**^⊥^	**ΔΔG° = -8.04**	**ΔΔH° = -10.6**	**ΔΔS° = -8.59**	**-TΔΔS° = 2.56**
**ITC**	**ΔG° = -9.6± 0.0**	**ΔH° = -11.8 ± 0.0**	**ΔS° = -6.7**	**-TΔS° = 2.2 ± 0.0**

^**⊥**^Overall reaction thermodynamics were obtained by summation of transition states (ΔΔG = ΣΔG_Forward_-ΣΔG_Reverse_). For example, ΔΔG° = ΔG(‡1,k_1_)—ΔG(‡1,k_2_) + ΔG(‡2,k_3_)—ΔG(‡2,k_4_).

## Discussion

To dissect the role of the *Pf*AMA1 DII loop in regulating RON2 binding, we engineered a construct of *Pf*AMA1 with the 37 residues DII loop [351–387] replaced with a shortened Gly-Ser linker of 7 residues. Based on the structural data, the ΔDII-*Pf*AMA1 construct is expected to mimic the displaced DII loop form of *Pf*AMA1 to present a mature ligand binding groove. Despite a similar structural architecture, *Pf*AMA1 has not been shown to present cross genera RON2 binding as has been observed for *Tg*AMA1 proteins [33]. Recently, Parker and Boulanger [25] proposed that the *Tg* DII loop acts as a structural gatekeeper by a mechanism of competitive binding with RON2. This mechanism enables the selective filtering of ligands that would not promote the functional AMA1-RON2 binary complex required for successful invasion of host cells. This mechanism of competitive binding is even more effective if the DII loop binds to the hydrophobic groove with a high affinity. However, *Pf* and *Tg* AMA1 differ markedly in the length and the mobility of their DII loop. *Tg*AMA1 DII loop is 13 amino acids shorter than *Pf*AMA1 DII loop and structural studies showed a tighter binding of the *Tg* DII loop in the ligand binding groove than for *Pf*AMA1 DII loop. The higher mobility of the *Pf*AMA1 DII loop is also supported by the lack of electron density in several crystal structures (PDB ID 2Q8B [11] and 4R1A [19]), by conformational exchange in ^19^NMR studies [34] and in molecular dynamics simulations [19]. As a result, a fast interconversion between a bound and an unbound *Pf* form of the DII loop is presumable, but only the unbound form gives access to an open binding site to *Pf*RON2sp1. The initial and final states for both *Pf* and ΔDII-*Pf* complexes are expected to be structurally similar [25] and both showed a similar thermodynamic profile for the free energy ΔΔG° (Fig 2). In addition, both *Pf*AMA1 and ΔDII-*Pf*AMA1 possess a conserved apical groove that is surrounded by six loops from domain I (loops Ia to If), but these are expected to undergo the same structural rearrangements upon *Pf*RON2sp1 binding. Thus, the observed differences between the two complexes are likely to be imparted primarily by the DII loop.

To better understand the role of the *Pf* DII loop upon RON2 binding, we investigated the kinetics and the thermodynamics of the interaction between *Pf*RON2sp1 and both native and ΔDII-loop forms of *Pf*AMA1. Kinetic reactions and K_d_ titration measurements were tracked by fluorescence anisotropy. For this purpose, we used of the previously described peptide *Pf*RON2sp1 (*Pf*RON2 segment [2021–2059]) [23] attached to the fluorescein probe 5-FAM (Table 1). The affinity values (K_d_) calculated from titrations (Fig 1) were in close agreement with those obtained by SPR studies [23] and ITC (Fig 2), yielding a K_d_ of 42 nM and 94 nM for *Pf*AMA1 and ΔDII-*Pf*AMA1, respectively (Table 2) and thus validating the reliability of the fluorescence anisotropy method. We next measured the kinetics of *Pf*AMA1-*Pf*RON2sp1 interaction to determine the rate constants k_on_ and k_off_ as a function of temperature (Fig 3) and concentration of *Pf*AMA1 (Fig 4). As the K_d_ is the ratio of the k_off_ and k_on_, determining these values may reveal the differences of the association and dissociation kinetics of *Pf*AMA1 and ΔDII-*Pf*AMA1 with *Pf*RON2sp1. Indeed, the preliminary analyses of the kinetic traces showed that the binding to ΔDII-*Pf*AMA1 was only marginally faster than to *Pf*AMA1. However, the rate of dissociation of the peptide from ΔDII-*Pf*AMA1 was considerably faster (Fig 3B and 3D and S1 Table), resulting in an overall weaker affinity whereas the DII loop in *Pf*AMA1 apparently slowed the dissociation event. These dissociation experiments were carried out at high excess of unlabeled *Pf*RON2sp1 (250× and 500×) against *Pf* and ΔDII-*Pf* complexes (Fig 4C and 4D) and were not affected by increasing unlabeled RON2 peptide consistent with a unimolecular mechanism. Thus, the labeled bound *Pf*RON2sp1 must first spontaneously leave the binding site before accommodating the unlabeled *Pf*RON2sp1.

**Fig 7:**
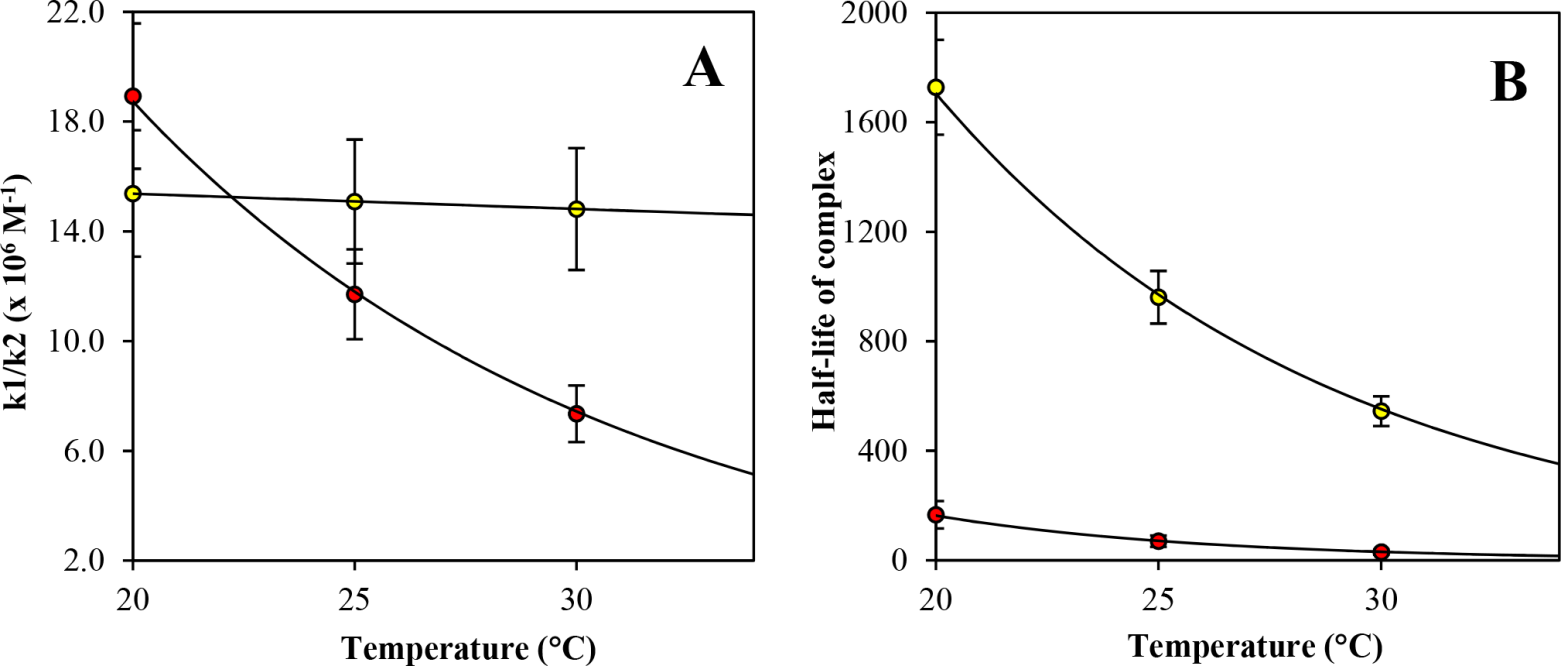
Temperature-dependence of A) the ratio of rate constants k1/k2. The presence of the DII loop made the first rate constant ratio less affected by temperature changes, as seen for *Pf*AMA1-F*PfRON2sp1 (yellow) in contrast with the slope observed for ΔDII-*Pf*AMA1-F*PfRON2sp1 (red). B) Temperature dependence of the complex half-life which is defined as the time required for 50% dissociation of the complex. At higher temperatures, the half-life decreased markedly. Error bars represent one standard deviation in the fluorescence-based experiments.

To further investigate the detailed binding mechanism, we carried out a global fitting integrating association, dissociation and titration equilibrium data into a model representing the simplest most probable reaction mechanism. The strongly biphasic kinetics (a fast and a slow phase) of both *Pf* complexes indicated a stepwise binding mechanism. Assessment of three different mechanisms resulted in a meaningful AIC value that favored the one-intermediate model (Table 3 and Fig 5). Based on this model, we then evaluated the respective rate constants (Table 4), complex half-life (Table 5) as a function of temperature and the thermodynamic profile (Table 6) and compared them to experimental results. At 25°C, the solid lines in Fig 3 represented the best fit of the experimental data. It is noteworthy that similar biphasic curves have been also observed in SPR experiments [35]. The affinity values (K_d_) calculated from the globally fitted rate constants (k_1_, k_2_, k_3_ and k_4_, Table 4) were in close agreement with those obtained by other experiments (Table 2) as were the experimental and calculated complex half-life values (Table 5). The complex half-life decreased markedly with increasing temperature for both complexes but showed a very short value of 30 seconds at 30°C (and even a predicted value of 10 s at 37°C) for ΔDII-*Pf* complex as compared to the 9 minutes for *Pf* complex (Table 5 and Fig 7B).

**Fig 8:**
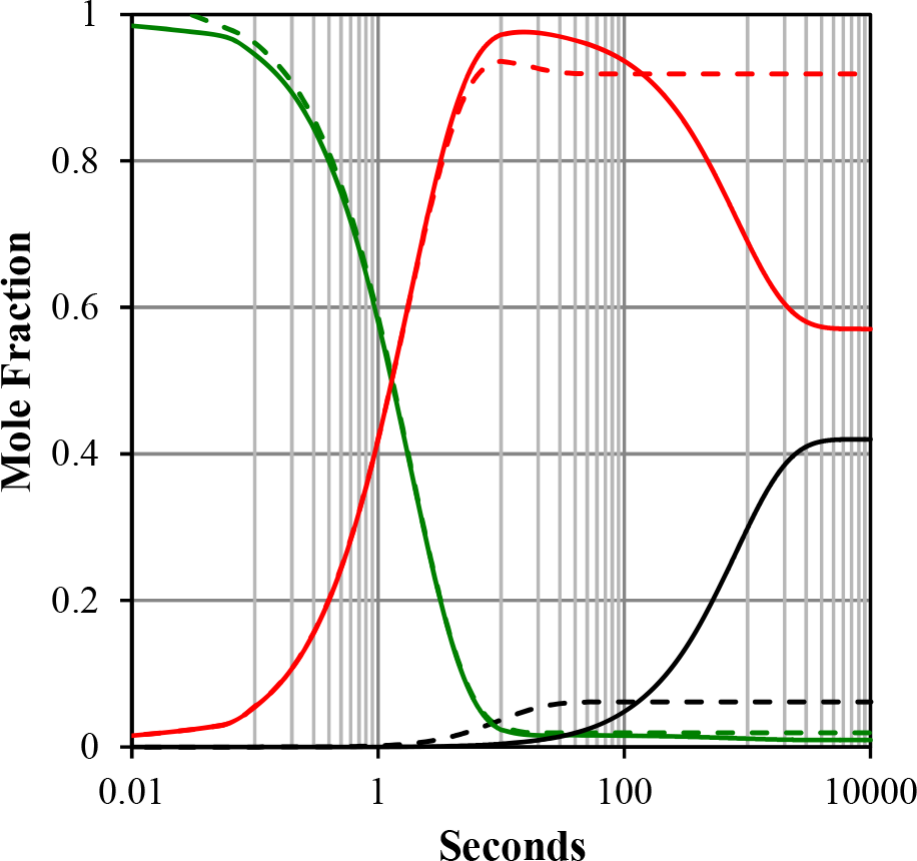
Reaction simulation of *Pf*AMA1 (solid lines) and ΔDII-*Pf*AMA1 (slashed lines) at 5 μM reacting with 1 μM of *Pf*RON2sp1 at 25°C. *Pf*RON2sp1 is colored in green, Intermediate is colored in red and final complex is colored in black. The lack of the DII loop is predicted to decrease markedly the molar fraction of the final complex (black slashed lines) as compared to the native one (solid black lines). It is also noteworthy that the maximum complex concentration for ΔDII-*Pf*AMA1 is obtained in less than 100 seconds whereas it needs more 400 seconds for *Pf*AMA1.

In the proposed model, the k_1_/k_2_ ratio for *Pf*AMA1, which is the first step of the reaction, was less affected by the temperature contrary to the modified ΔDII-*Pf*AMA1 (Table 4 and Fig 7A). As the rate constant k_1_ increased with higher temperature, this indicated that the reverse rate constant k_2_ increased proportionally for *Pf*AMA1 but increased even more for ΔDII-*Pf*AMA1. As well, rate constants k_3_ and k_4_ were 1 to 2 orders of magnitude faster for ΔDII-*Pf*AMA1 than for *Pf*AMA1 indicating markedly different kinetics of the second step of the reaction mechanism and suggesting that the DII loop affected strongly the release kinetics of the second step (k_4_). Indeed, based on this model, the simulation of the molar fraction of species as a function of time showed that the final ΔDII-*Pf*AMA1 complex is predicted to be only 6% in comparison with 42% for the *Pf*AMA1 complex at 25°C (Fig 8). Interestingly, we also could observe that the intermediate species predominates but that the formation of the final complex is faster for the ΔDII-*Pf*AMA1 form (less than 100 s).

The calculated thermodynamic profile indicated that the intermediate state was entropically driven in the case of *Pf*AMA1, likely favored by the displacement of the DII loop and consequently not observed for the ΔDII-*Pf*AMA1 form. It also indicated that, in this first step, the DII loop eased the releasing of the bound peptide in *Pf*AMA1 because enthalpy values of activation $\Delta H_{forward}^{‡1}$ and $\Delta H_{reverse}^{‡2}$ were almost equivalent (~15.4 and 16.1 kcal/mol, respectively (Table 6)) whereas in ΔDII-*Pf*AMA1, these values were very different and implied that the reverse movement (to the initial unbound state) was more costly (13.4 and 30.1 kcal/mol, respectively (Table 6)). Thus, during the first step, the DII loop may act as a gatekeeper that would displace a low affinity ligand, for example another apicomplexa RON2 peptide, by a mechanism of competitive binding whereas in the case of the binding of its cognate *Pf*RON2, the DII loop kinetically locks the functional binary complex that results from the second step of the reaction.

**Fig 9:**
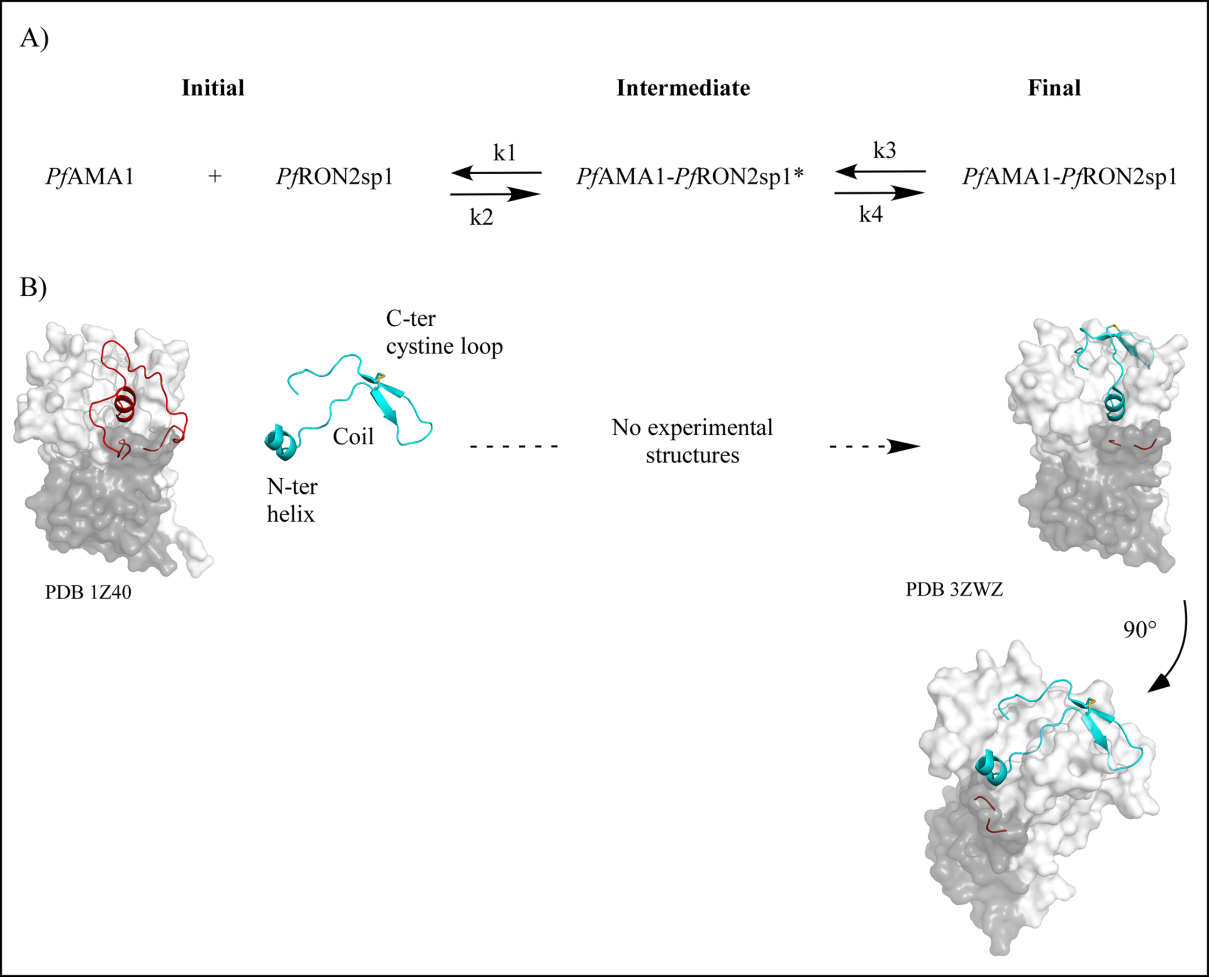
Known 3D Structures. **A)** Two-step reaction mechanism with one intermediate of *Pf*RON2sp1 peptide binding to *Pf*AMA1. **B)** The apo structure of native *Pf*AMA1 (PDB code 1Z40) is shown in surface representation (domain DI is colored in white and domain DII is colored in grey). The DII loop is in ribbon representation and colored in red. *Pf*RON2sp1 is in ribbon representation and is colored in cyan and the disulfide bridge is colored in yellow. RON2 peptides are composed of three parts: a N-terminal helix, a coil and a disulfide-closed β-hairpin loop (also called cystine loop). In holo *Pf*AMA1 (final complex), the displaced DII loop is not visible in the structure PDB code 3ZWZ. Co-structures showed that the N-terminal helix of *Pf*RON2sp1 seated at one end of the AMA1 apical groove in place of the DII loop and extended through an ordered coil to the β-hairpin loop that bound the other end of the apical groove at the opposite of the DII loop [23].

Different structural mechanisms could potentially describe the one-intermediate model with an initial encounter complex associated with rate constants k_1_ and k_2_ followed by the conversion of the intermediate complex into its final conformation (rate constants k_3_ and k_4_). Structural and Ala-scan results formerly suggested a stepwise binding mechanism where contact was initiated between the end of the AMA1 and the cystine loop followed by the binding of the helix part [21, 23]. In such a model, rate constants k_3_ and k_4_ would represent a conformational change of *Pf*RON2sp1. Indeed, RON2 peptides are composed of three parts, a N-terminal helix, a coil and a disulfide-closed β-hairpin loop (also called cystine loop) (Fig 9) that possess some flexibility when compared to one another as suggested molecular dynamic studies (data not shown). However, it is important to point out that these kinetic experiments do not allow the indentification of the intermediate states. It must also be clarified that the present experimental data and models provide a rationale for an *in vitro* reductionist system far from physiological conditions and where *Pf*RON2sp1 is a peptide extracted from the ectodomain of the 2189 residues membrane protein *Pf*RON2 that may impact the flexibility of the extracellular domain *in vivo*. Further biophysical experiments will be required to follow the binding dynamics of *Pf*AMA1 and *Pf*RON2sp1 and to examine at the same time the kinetics of both the DII loop and the RON2 peptide (for example by using FRET experiments).

## Conclusion

In conclusion, we investigated the kinetics and the thermodynamics of the interaction of *Pf*RON2sp1 with *Pf*AMA1 and ΔDII-*Pf*AMA1. The experimental data are consistent with an new influential role for the DII loop in kinetically locking the functional binary *Pf*AMA1-*Pf*RON2sp1 complex. Although the DII loop thermodynamically stabilizes the binding of *Pf*RON2sp1 by a 2-fold larger affinity, we showed that the main effect is observed on the kinetics by restraining the release of the *Pf*RON2 peptide and increasing the complex half-life by 18-fold with a value of 900 s (15 min) at 25°C for *Pf* complex in comparison with 48 s for ΔDII-*Pf* complex. Interestingly, the complex half-life of the *Pf*AMA1-*Pf*RON2sp1 complex is compatible with the time frame of erythrocyte invasion by *Plasmodium falciparum* merozoites [36–38]. Our data reveals a more comprehensive model of invasion in which the DII loop may be a key regulatory feature that determines the stability of the MJ.

## Supporting Information

S1 Fig Fluorescence change over time. (PDF)

S2 Fig Global fitting when assessing a simple reversible reaction model. (PDF)

S3 Fig Global fitting when assessing an interconversion model. (PDF)

S1 Table Association and dissociation curves fitted by a double-exponential function. (PDF)

S1 Text Fluorescence-based experiments. (PDF)

S2 Text Titration experiments. (PDF)

S3 Text Model Evaluation. (PDF)

## Abbreviations

AMA1: Apical Membrane Antigen 1
RON2: Rhoptry Neck Protein 2
*Pf* complex: *Pf*AMA1-F**Pf*RON2sp1
ΔDII-*Pf* complex: ΔDII-*Pf*AMA1-F**Pf*RON2sp1
