# Supplementary material for: Stability of the Plasmodium falciparum AMA1-RON2 Complex Is Governed by the Domain II (DII) Loop
Source: PLoS One. 2016 Jan 5;11(1):e0144764. doi: 10.1371/journal.pone.0144764 (PMC4701444; doi:10.1371/journal.pone.0144764)
Supplement: S1 Fig — (PDF) [file pone.0144764.s001.pdf]

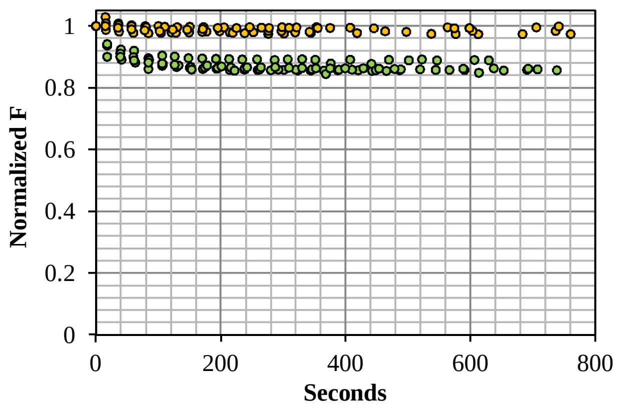

S1 Figure. Fluorescence change over time for ΔDII-*Pf*AMA1 (gold) and *Pf*AMA1 (green) at concentration of 50 nM reacting with 10 nM of F**Pf*RON2sp1.
