# Supplementary material for: Stability of the Plasmodium falciparum AMA1-RON2 Complex Is Governed by the Domain II (DII) Loop
Source: PLoS One. 2016 Jan 5;11(1):e0144764. doi: 10.1371/journal.pone.0144764 (PMC4701444; doi:10.1371/journal.pone.0144764)

S3 Figure. Global fitting when assessing an interconversion model: $(A_{Inactive} \underset{k2}{\overset{k1}{\leftrightarrow}} A^{*}_{Active} + B \underset{k4}{\overset{k3}{\leftrightarrow}} C)$. The solid black lines are the globally fitted data according to an interconversion model (eqn 5) for *Pf*AMA1-F**Pf*RON2sp1 **(A, B)** and ΔDII-*Pf*AMA1-F**Pf*RON2sp1 **(C, D)** at 20 °C (blue), 25 °C (yellow) and 30 °C (red).

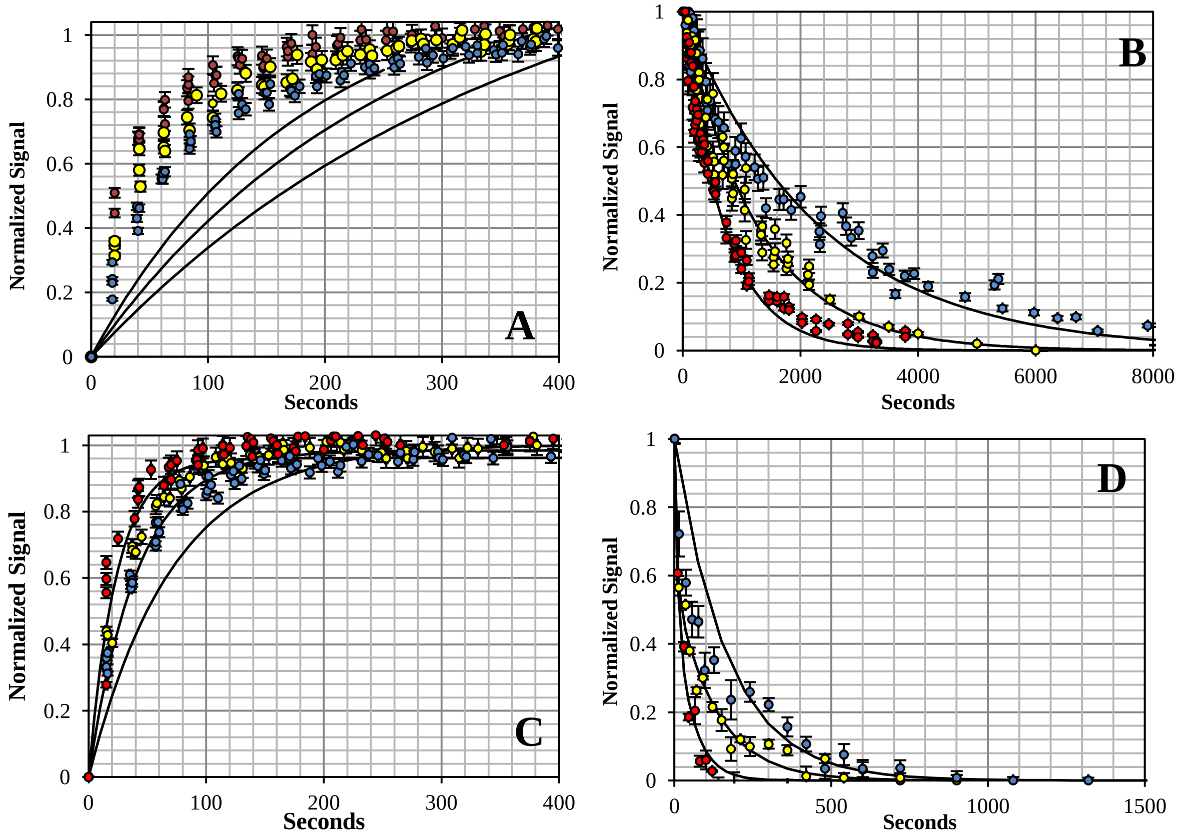

Supplement: S3 Fig — (PDF) [file pone.0144764.s003.pdf]
