# Supplementary material for: Stability of the Plasmodium falciparum AMA1-RON2 Complex Is Governed by the Domain II (DII) Loop
Source: PLoS One. 2016 Jan 5;11(1):e0144764. doi: 10.1371/journal.pone.0144764 (PMC4701444; doi:10.1371/journal.pone.0144764)
Supplement: S1 Table — (PDF) [file pone.0144764.s004.pdf]

S1 Table. Association and dissociation curves fitted by a double-exponential function: $\alpha_1 exp(-\lambda_1 * t) + \alpha_2 exp(-\lambda_2 * t)$ with two pre-exponentials ($\alpha 1$ and $\alpha 2$) and two eigenvalues (λ). These values can be used to generate the observed raw data but these fits are not the ones that have been calculated when modeling the reaction mechanism.

***Pf*AMA1-*Pf*RON2sp1**		**20 °C**	**25 °C**	**30 °C**
Association *Pf*AMA1 (50 nM)	α_1=	-0.6295 (±0.02664)	-0.6350 (±0.0321)	-0.7171 (±0.0438)
	λ_1=	0.0210 (±0.0030)	0.0320 (±0.0029)	0.0382 (±0.0038)
	α_2=	-0.3705 (±0.0152)	-0.3650 (±0.0366)	-0.2829 (±0.0467)
	λ_2=	0.0042 (±0.0005)	0.0060 (±0.0010)	0.0100 (±0.0010)
Association *Pf*AMA1 (100 nM)	α_1=	-0.5323 (±0.0157		
	λ_1=	0.045 (±0.0030)		
	α_2=	-0.4677 (±0.0122)		
	λ_2=	0.0075 (±0.0005)		
Association *Pf*AMA1 (200 nM)	α_1=	-0.1799 (±0.0198)		
	λ_1=	0.0150 (±0.0010)		
	α_2=	-0.8201 (±0.028)		
	λ_2=	0.0800 (±0.0010)		
Dissociation	α_1=	0.2675 (±0.0578)	0.1987 (±0.0759)	0.1599 (±0.0236)
	λ_1=	0.00214 (±0.0007)	0.0035 (±0.0007)	0.0050 (±0.0008)
	α_2=	0.7325 (±0.0613)	0.8013 (±0.0550)	0.8401 (±0.0134)
	λ_2=	0.0003 (±0.00003)	0.00063 (±0.0001)	0.0012 (±0.0001)
**ΔDII-*Pf*AMA1-*Pf*RON2sp1**		**20 °C**	**25 °C**	**30 °C**
Association ΔDII-*Pf*AMA1 (50 nM)	α_1=	-0.2424 (± 0.0720)	-0.7735 (± 0.0240)	-0.4853 (±0.0600)
	λ_1=	0.0087 (± 0.0016)	0.0369 (± 0.0065)	0.0779 (±0.0100)
	α_2=	-0.7576 (± 0.0710)	-0.2264 (± 0.0240)	-0.5147 (±0.0600)
	λ_2=	0.0325 (± 0.0029)	0.0149 (± 0.0067)	0.0306 (±0.0149)
Association ΔDII-*Pf*AMA1 (100 nM)	α_1=	-0.3016 (± 0.0349)		
	λ_1=	0.015 (± 0.0020)		
	α_2=	-0.6984 (± 0.0498)		
	λ_2=	0.0927 (± 0.0122)		
Association ΔDII-*Pf*AMA1 (200 nM)	α_1=	-0.0999 (± 0.0900)		
	λ_1=	0.030 (± 0.0050)		
	α_2=	-0.9001 (± 0.0900)		
	λ_2=	0.2248 (± 0.0200)		
Dissociation	α_1=	0.5449 (±0.0390)	0.4812 (±0.0502)	0.5239 (±0.0851)
	λ_1=	0.0032 (±0.0003)	0.0060 (±0.0009)	0.0180 (±0.0008)
	α_2=	0.4551 (±0.0460)	0.5115 (±0.0633)	0.4849 (±0.0800)
	λ_2=	0.0451 (±0.0105)	0.0906 (±0.008)	0.1200 (±0.00010)
