# Supplementary material for: Stability of the Plasmodium falciparum AMA1-RON2 Complex Is Governed by the Domain II (DII) Loop
Source: PLoS One. 2016 Jan 5;11(1):e0144764. doi: 10.1371/journal.pone.0144764 (PMC4701444; doi:10.1371/journal.pone.0144764)
Supplement: S1 Text — (PDF) [file pone.0144764.s005.pdf]

S1 Text. Fluorescence-based experiments. Anisotropy (r, eqn S1-1) and polarization (P, eqn S1-2) fluorescence methods measure the rotational mobility of the dye and labeled biomolecule.[1] Anisotropy in contrast to polarization is normalized by the total fluorescence intensity (eqn S1-3). The term G (eqn S1-4) is the correction factor due to differences in sensitivities of the photomultiplier tube (PMT) to vertical or horizontal light. In our instrument, the excitation light is provided by a xenon arc lamp filtered through an excitation polarizer and the emission signal is collected by an analyzer set at vertical $I_{\parallel}$ or horizontal position $I_{\perp}$.

$$r = \frac{I_{\parallel} - G * I_{\perp}}{I_{\parallel} + 2\, G * I_{\perp}} \qquad \text{eqn S1-1}$$

$$P = \frac{I_{\parallel} - G * I_{\perp}}{I_{\parallel} + G * I_{\perp}} \qquad \text{eqn S1-2}$$

$$F = I_{\parallel} + 2\, G * I_{\perp} \qquad \text{eqn S1-3}$$

where

$$G = \frac{I_{\parallel}}{I_{\perp}} \qquad \text{eqn S1-4}$$

$$r(t) = {\sum x_i(t) * QY_i * r_i}\Big/{F(t)} \qquad \text{eqn S1-5}$$

$$rF(t) = \sum x_i(t) QY_i r_i \qquad \text{eqn S1-6}$$

Anisotropy is preferred as it correctly tracks the molecular species formation when fluorescence changes are small [2]. However, if the change in fluorescence is significant (e.g. fluorescence quenching), the reactions have to be followed by the product of the two signals: *rF(t)* (eqn S1-6) in order to track the chemical species (x_i) and the respective quantum yields (*QYi*) to elucidate the reaction mechanism. In practice, if F is constant or very small (less than 25%), the *r* and *rF* signals are almost equivalent, and *r* can be used to simplify the analysis. The fluorescence change of ΔDII-*Pf*AMA1 and *Pf*AMA1 reacting with the F**Pf*RON2sp1 showed very little fluorescence quenching (S1 Fig).

References

1. Lakowicz, J. R., *Principles of Fluorescence Spectroscopy* 3rd ed.; Springer Singapore, p 353-358, 2006; p 954.

2. Otto, M. R.; Lillo, M. P.; Beechem, J. M., Resolution of multiphasic reactions by the combination of fluorescence total-intensity and anisotropy stopped-flow kinetic experiments. *Biophys. J.* **1994,** 67, (6), 2511-2521.
