# Supplementary material for: Stability of the Plasmodium falciparum AMA1-RON2 Complex Is Governed by the Domain II (DII) Loop
Source: PLoS One. 2016 Jan 5;11(1):e0144764. doi: 10.1371/journal.pone.0144764 (PMC4701444; doi:10.1371/journal.pone.0144764)
Supplement: S2 Text — (PDF) [file pone.0144764.s006.pdf]

S2 Text. Titration experiments. Association constant K_a was calculated by solving the protein fraction saturation by a quadratic equation as following:

$$K_a = \frac{[PX]}{[P][X]} \qquad \text{eqn S2-1}$$

Where [PX] is the complex with labeled ligand, [P] and [X] are the free protein and free ligand. Therefore, the total protein [P_T] and total Ligand [X_T] are defined as:

$$P_T = P + PX \qquad \text{eqn S2-2}$$

$$X_T = X + PX \qquad \text{eqn S2-3}$$

The fraction protein bound is:

$$Y = \frac{[PX]}{[P_T]} = \frac{[PX]}{[P]+[PX]} \qquad \text{eqn S2-4}$$

Likewise in terms of labeled ligand saturation is

$$Y = \frac{[PX]}{[X_T]} = \frac{[PX]}{[X]+[PX]} \qquad \text{eqn S2-5}$$

Substituting PX from eqn S2-1 into eqn S2-5

$$Y = \frac{K_a[P][X]}{[X]+K_a[P][X]} = \frac{K_a[P]}{1+K_a[P]} \qquad \text{eqn S2-6}$$

We substitute P from eqn S2-2 into eqn S2-6

$$Y = \frac{K_a[P_T-PX]}{1+K_a[P_T-PX]} \qquad \text{eqn S2-7}$$

From eqn S2-5 we get $[PX] = Y[X_T]$

$$Y = \frac{K_a\{[P_T]-Y\,[X_T]\}}{1+\,K_a\{[P_T]-Y\,[X_T]\}} \qquad \text{eqn S2-8}$$

Eqn S2-8 is a quadratic equation, defined as following:

$$a\boldsymbol{x}^{\boldsymbol{2}} + b\boldsymbol{x} + c = 0 \qquad \text{eqn S2-9}$$

Rearranging eqn S2-8:

$$Y + K_aP_TY - Y^2X_TK_a = K_aP_T - K_aX_TY \qquad \text{eqn S2-10}$$

$$0 = X_TK_a\boldsymbol{Y}^{\boldsymbol{2}} - (1 + K_aP_T + K_aX_T)\boldsymbol{Y} + K_aP_T \qquad \text{eqn S2-11}$$

Solving the quadratic equation:

$$\boldsymbol{Y} = \frac{-b \pm \sqrt{b^2 - 4ac}}{2a}$$ eqn S2-12

Where

$a = X_T K_a$, $b = -(1 + K_a P_T + K_a X_T)$, $c = P_T K_a$
