# Supplementary material for: Stability of the Plasmodium falciparum AMA1-RON2 Complex Is Governed by the Domain II (DII) Loop
Source: PLoS One. 2016 Jan 5;11(1):e0144764. doi: 10.1371/journal.pone.0144764 (PMC4701444; doi:10.1371/journal.pone.0144764)
Supplement: S3 Text — (PDF) [file pone.0144764.s007.pdf]

S3 Text. Model Evaluation. The simplex optimization algorithms aim to minimize the residual sum of squared (RSS) (eqn S4-1) of observed versus calculated data points (n) for each data set (association, dissociation and equilibrium at 3 temperatures and 3 concentrations in the case of the association set) (eqn S4-2). In our case, the Global Residual Sum of Square (RSS_{Global}) was calculated with 3 equilibrium, 5 association and 3 dissociation data sets that gave of total number of 11 curves to fit.

$$RSS = \sum_{i=1}^{n} \frac{(observed_i - calculated_i)^2}{n} \quad \text{eqn S4-1}$$

$$RSS_{Global} = \frac{\sum_1^5 RSS_{Association} + \sum_1^3 RSS_{Dissociation} + \sum_1^3 RSS_{Equilibrium}}{N} \quad \text{eqn S4-2}$$

The best model was selected by using the Akaike selection criterion (AIC) (eqn S4-3) which estimates the model quality and enables to compare models [1]. The AIC value is a powerful model discriminator that takes into account for the RSS and the number of estimated parameters *k'* (e.g rate constants and activation energies in our models) [2]. The second term *k'* provides a penalty to avoid overfitting when the number of parameters increased in the model. The model with the lowest error levels is the most likely.

$$AIC = NLog\left(\frac{RSS_{Global}}{N}\right) + 2k' \quad \text{eqn S4-3}$$

References

1. Akaike, H., A new look at the statistical model identification. *Ann. Inst. Stat Math.* **1973,** 22, 203-217.
2. Burnham, K. P.; Anderson, D. R., *Model Selection and Multimodel Inference: A Practical Information-Theoretic Approach*. 2nd ed. ed.; Springer-Verlag: 2002.
